# Design of Value Chains for Microalgal Biorefinery at Industrial Scale: Process Integration and Techno-Economic Analysis

**DOI:** 10.3389/fbioe.2020.550758

**Published:** 2020-09-08

**Authors:** Petronella M. Slegers, Giuseppe Olivieri, Elke Breitmayer, Lolke Sijtsma, Michel H. M. Eppink, Rene H. Wijffels, Johannes H. Reith

**Affiliations:** ^1^Biobased Chemistry and Technology, Wageningen University & Research, Wageningen, Netherlands; ^2^Nova-Institute for Ecology and Innovation, Hürth, Germany; ^3^Operations Research and Logistics, Wageningen University & Research, Wageningen, Netherlands; ^4^Bioprocess Engineering – AlgaePARC, Wageningen University & Research, Wageningen, Netherlands; ^5^Dipartimento di Ingegneria Chimica, dei Materiali e della Produzione Industriale, Università degli Studi di Napoli Federico II, Naples, Italy; ^6^Wageningen Food & Biobased Research, Wageningen University & Research, Wageningen, Netherlands; ^7^Faculty of Biosciences and Aquaculture, Nord University, Bodø, Norway

**Keywords:** microalgae, biorefinery, value chain, process design, techno-economic evaluation, revenue

## Abstract

The objective of this work was to identify industrial scenarios for the most promising microalgal biorefinery value chains on the basis of product selection, yields, and techno-economic performance, using biological characteristics of algae species. The development, value creation, and validation of several new processing routes with applications in food, aquafeeds and non-food products were particularly considered in this work. The techno-economic performance of various single product value chains (SP) and multiproduct value chains (MP) was evaluated for four industrial microalgal strains. Cost-revenue optimization was done for a 10 kton microalgal dry weight y^–1^ simulated biorefinery plant, using flow sheeting software for equipment sizing, mass and energy flow modeling, and subsequent techno-economic evaluation. Data on yield, material and energy consumption were based on pre- and pilot size production plants (TRL 5–6). Revenue optimization was accomplished by first analyzing the performance of single product value chains of the microalgal strains. Subsequently, a strategy was developed to exploit almost all biomass based on the most promising microalgal strains. The cultivation costs are most of the time the major costs of the value chains. For the single product value chains common process bottlenecks are low product yields, especially for soluble proteins where only a small fraction of the biomass is leading to economic value. The biorefinery costs (excluding cultivation) vary significantly for various species, due to the species-specific operating conditions as well as differences in product yields. For the evaluated single product value chain scenarios the costs for utilities and other inputs were in general the highest contributing expenses. A biorefinery approach significantly increases the biomass utilization potential to marketable products from 7–28% to more than 97%. Although the cascading approach increases the total production costs of the multiproduct value chains significantly, this is more than compensated by the increased overall biomass revenue. For all selected multiproduct chains there is a significant potential to become profitable at a relevant industrial scale of 10 kton per year. Additional insights in the product functionality, quality, and their market size are needed to narrow down the wide range of foreseen product revenues and resulting profits.

## Highlights

- Biomass cascading improves the biomass exploitation from < 25% to > 95%.

- Cultivation costs are most of the time the major costs of the value chains.

- Biorefinery costs vary significantly for various microalgal species.

- Profitable biorefinery processes have been developed at relevant pilot scale.

- Multiproduct biorefinery can enhance profits in the microalgae value chain.

## Introduction

Nowadays, the economy is changing from being fossil-based toward renewable and biobased. In a biobased economy, biomass is fully valorized and used for the sustainable production of food, feed, chemicals, fuels, power, and heat (International Energy Agency [IEA]2009)^1^. Microalgae have a huge potential as bioresource for food, (aqua)feed, chemicals, and materials. Microalgae have the advantage of high growth yields, low land requirements, and the ability to grow on salt water and waste water (Wijffels and Barbosa, 2010; Wijffels et al., 2010). Furthermore, microalgae contain various valuable components that can be processed into a versatile range of products (de la Noue and de Pauw, 1988; Buono et al., 2014), ranging from bulk product such as food commodities to specialty ingredients for food and non-food applications (Wijffels et al., 2010; Milledge, 2011; Draaisma et al., 2013; Ruiz et al., 2016). Well-known examples of microalgal products are pigments from *Dunaliella salina* and *Haematococcus pluvialis*, food supplements from *Chlorella* and *Spirulina*, and omega–3 rich oils from *Nannochloropsis gaditana, Schizochytrium* sp., and *Crypthecodinium cohnii*. A complete list of other potential products have been recently reviewed by Chew et al. (2017) and Chandra et al. (2019).

Currently, most applications of microalgal biomass are still for food, feed, and high value applications (Vandermeulen et al., 2012; Vigani et al., 2015; Rajesh Banu et al., 2020). The economic potential of various microalgae products has been studied previously by several authors (Davis et al., 2014; Gong and You, 2015; Quinn and Davis, 2015; Dong et al., 2016; Thomassen et al., 2016; Laurens et al., 2017; Asiedu et al., 2018; DeRose et al., 2019). Ruiz et al. (2016) gave an overview of the different market scenarios for a benchmark microalgal composition. The cost estimates for cultivation (including harvesting) are in the range of 3.20–11.00 € kg^–1^ biomass (100 ha cultivation system; Ruiz et al., 2016). Processing costs (excluding cultivation and harvesting) were estimated between 0.40–1.80 € kg^–1^ biomass for commodities and 2.30–4.30 € kg^–1^ biomass for speciality products in a biorefinery (both designed for 100 ha production scale; Ruiz et al., 2016). The cost estimates for biofuel production are currently ranging from an optimistic 0.55 to 9.00 € L^–1^ biodiesel corresponding to about 1.65–27.00 € kg^–1^ microalgal biomass (Quinn and Davis, 2015). The production of high value products shows more favorable economics. For omega-3 fatty acids, the production costs ranged between 2.35 and 8.10 € kg^–1^ microalgal biomass (based on 100 ha production scale) depending on the production system and location (Chauton et al., 2015). Pigment production from microalgae is also economically viable (Ruiz et al., 2016; Thomassen et al., 2016) with costs ranging from 12.50 to 107.95 € kg^–1^ biomass, depending on the cultivation and process technologies used, as well as type of pigments for market applications.

A biorefinery approach can be used to valorize all valuable biomass components by using a combination of several separation techniques and potentially leads to better economic performance (Eppink et al., 2019). The overall biorefinery costs (including cultivation and harvesting) are known to strongly depend on the cultivation location, cultivation system, biomass composition, and selected downstream processing technologies (Norsker et al., 2011; Delrue et al., 2013; Draaisma et al., 2013; Chauton et al., 2015; Quinn and Davis, 2015; Ruiz et al., 2016). Beal et al. (2015) provided an extensive techno-economic analysis of an energy-production focussed biorefinery chain (cultivation and downstream processing), including a diverse product portfolio (biodiesel, biocrude, animal feed, and ethanol), several process scenarios, two microalgal strains, two locations, and two cultivation conditions at 1 ha scale and projected to 100 ha scale. The scenario for commodities such as fuels is the least favorable option (Beal et al., 2015). Ruiz et al. (2016) and Beal et al. (2018) have extended the techno-economic analysis to more complete biorefineries, including various production scenarios associated to wider product portfolios (fuel, food and feed commodities, food additives, cosmetics, and pharma) and a larger variety of locations (Spain, Saudi Arabia, Netherlands, Turkey, Hawaii, and Thailand).

The recent analyses performed by Beal et al. (2015); Ruiz et al. (2016), and Beal et al. (2018) on the economics of the biorefinery are still limited by: (1) extrapolation of data on separation performances obtained at lab-scale and then scaled up to 100 ha production scale; (2) estimation of production cost based on extrapolation of data obtained at pre-pilot scale (for example from AlgaePARC, 25 m^2^) to 1 and 100 ha scale; (3) analysis of potential market for microalgal components based on a survey of current commercially substitute products, (4) generic processing efficiencies that do not take into account the effect of biological characteristics of algae species (such as cell size, cell wall thickness, and biochemical composition) on the processing performance (Gupta et al., 2017; de Carvalho et al., 2020). Additionally, commercial implementation of microalgal biorefineries is limited due to: (1) a still unfavorable balance between costs and revenues, (2) lack of well-established biorefinery processes at relevant industrial scale, which have to be tailored to the features of the specific microalgal components, (3) lack of available, validated market applications for the microalgal components.

In the EU FP7 MIRACLES-project these issues were addressed by developing novel biorefinery processes incorporating mild cell disruption and environmentally sustainable extraction and fractionation processes, and by proofing the commercial viability of the products by testing their functionality and formulations based on established industrial microalgal strains and potential business and end-users (partners of the project: Chimar Hellas AE, CropEye, DSM Food specialties BV, EcoTreasures BVBA, Ewos innovations AS, Fitoplancton Marino SL, ImEnz Bioengineering BV, Natac biotech SL, Rodenburg Biopolymers BV, Sparos LDA, Unilever Research and Development Vlaardingen BV, Value for Technology BVBA).

The objective of this work is to identify industrial product scenarios for the most promising microalgal biorefinery value chains on the basis of product selection, product yield and techno-economic performance using biological characteristics of algae species. In particular, the development, value creation, and validation of several new processing routes with product applications in food, aquafeeds and non-food products were considered in this work. The chains are designed using processing techniques developed or improved in the MIRACLES-project, complemented with data from best practice techniques (when required) and based on the marketable products that were validated in the project. The techno-economic performance of various single and multiproduct value chains was evaluated for four benchmark industrial microalgal strains.

## Materials and Methods

First single product value chains were selected and for each chain a processing chain was designed and implemented in SuperPro Designer^®^. The processing chains integrate novel technologies with benchmark technologies, both developed and tested within the framework of the MIRACLES-project. A sensitivity analysis was applied to determine the range of processing cost. After economic evaluation of the single product value chains, four multiproduct value chains were designed and evaluated, taking advantage of a cascading principle and thereby valorizing almost all biomass.

### Single Product Value Chains

Five single product value chains were selected and analyzed for identifying the most suitable microalgal biorefineries. Single product value chains are those that have one main valuable marketable product, the remainder of the biomass being regarded as residue for lower value applications. Four benchmark microalgal strains have been adopted: *Nannochloropsis gaditana* (marine), *Scenedesmus obliquus*^2^ (freshwater), *Phaeodactylum tricornutum* (marine), and *Isochrysis galbana* (marine). A large variety of interesting products is reported for these strains: polyunsatured fatty acids (C18:3, C20:5, and C22:6), sterols, functional proteins, carotenoids/antioxidants, and specialty carbohydrates (Pulz and Gross, 2004; Milledge, 2011). The choice was made for the following main products:

– whole microalgae (SP-I).
– broken microalgae (SP-II).
– water-soluble native proteins (SP-III).
– pigments dissolved in lipids (SP-IV).
– triacylglyceride-rich oil (SP-V).

**FIGURE 1:**
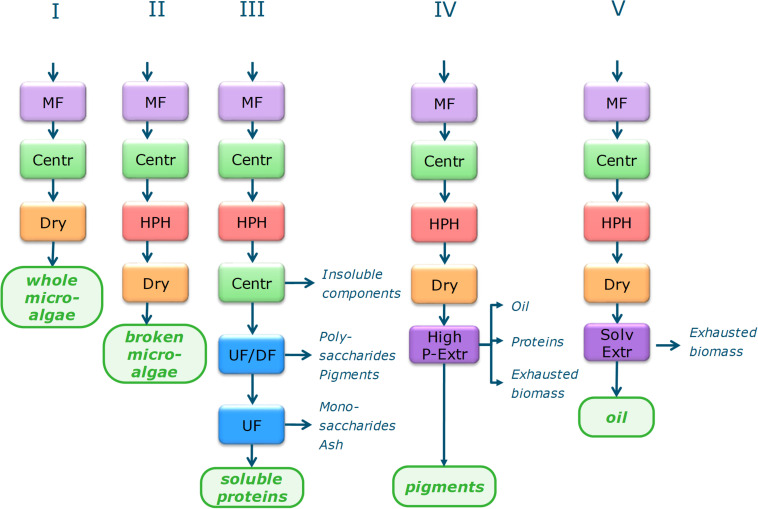
Biorefinery process schemes for single product value chains. MF, microfiltration; Centr, centrifugation; Dry, Spray drying; HPH, high pressure homogenization; UF, ultrafiltration; UF/DF, ultrafiltration/diafltration; High P–Extr, high pressure extraction (PLE, pressurized liquid extraction; GXL, gas expanded liquid extraction; SFE, supercritical fluid extraction), Alkal Extr, alkaline extraction; Enz Hydr, enzymatic hydrolysis.

A scheme of the five single product (SP) value chains is presented in Figure 1. For each of these products a process chain was designed and these designs were applied to all four microalgal strains, resulting in 20 possible production scenarios. Two cultivation conditions have been addressed in order to optimize the content of the main product to be extracted and purified: (1) nutrient replete cultivation conditions (‘N^+^-biomass’) and, (2) cultivation under nutrient limitation by nitrogen starvation to enhance the TAG content (‘N^–^-biomass’).

Each process chain starts with harvesting by microfiltration and centrifugation. In SP-I the biomass is dried to obtain the main product of whole microalgae. For the other SP chains cell disruption by high pressure homogenization is applied, followed by a drying step for SP-II, SP-IV, and SP-V. SP-III continues after disruption with two–step centrifugation with resuspension in water for separating the supernatant from cell debris, followed by UF/DF at 300 kDa (separation of polysaccharides from soluble proteins) and at 8 kDa (separation of soluble proteins from monosaccharides and ashes). SP-IV applies high pressure pigment extraction, the procedure is species dependent:

– *I. galbana*, spray drying, SFE→GXL→PLE
– *I. galbana*, no pre-processing, directly the reverse process PLE→GXL→SFE
– *N. gaditana*, disruption → spray drying → SFE→PLE
– *P. tricornutum*, disruption → spray drying → PLE
– *S. obliquus*, disruption → spray drying → SFE→GXL→PLE

Single product SP-V uses solvent extraction by a mixture of hexane with ethanol or isopropanol. Details on each process step are given in the Supplementary Material.

### Multiproduct Value Chains

**FIGURE 2:**
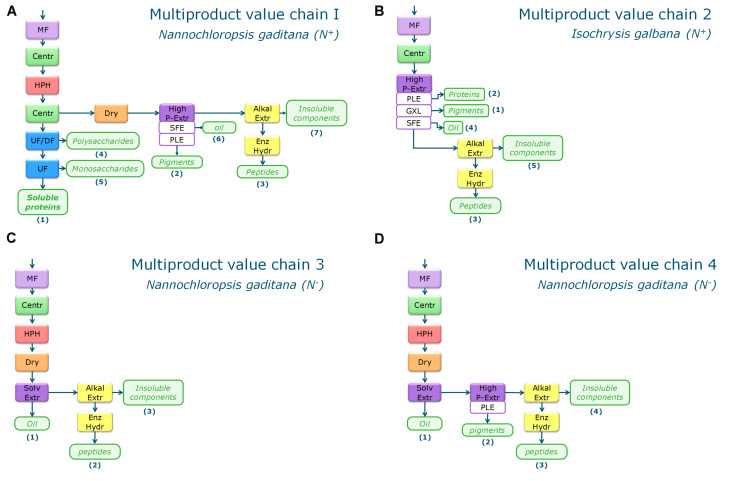
Biorefinery process schemes for the four multiproduct value chains **(A–D)**. MP Chain 1 **(A)** and 2 **(B)** are based on N^+^-biomass, MP chain 3 **(C)** and 4 **(D)** are using N^–^ -biomass. MF, microfiltration; Centr, centrifugation; Dry, Spray drying; HPH, high pressure homogenization; UF, ultrafiltration; UF/DF, ultrafiltration/diafltration; High P–Extr, high pressure extraction (PLE, pressurized liquid extraction; GXL, gas expanded liquid extraction; SFE, supercritical fluid extraction), Alkal Extr, alkaline extraction; Enz Hydr, enzymatic hydrolysis.

The multiproduct chains have multiple valuable marketable products. These multiproduct value chains (MP) were developed on basis of the most promising single product value chain scenarios. The biomass valorization was enhanced by applying a cascade approach to the multiproduct chains. All residue streams were either directly linked to a specific market application or were subjected to further refinery processing. Focus was on the main biomass components in terms of mass and/or product value. Figures 2A–D shows the flowsheets of the four selected multiproduct value chains. All MP chains start with harvesting by microfiltration and centrifugation.

The first multiproduct value chain (MP1) uses *N. gaditana* (‘N^+^-biomass’) and focusses on extraction of soluble proteins. The biorefinery processing consists of:

– Cell disruption by high pressure homogenization.
– Two-step centrifugation with resuspension in water for separating the supernatant from cell debris.
– UF/DF at 300 kDa (separation of polysaccharides from soluble proteins) and at 8 kDa (separation of soluble proteins from monosaccharides and ashes).
– Cell debris is dried and high-pressure extraction is applied to yield a pigment containing fraction.
– Alkaline extraction of proteins from the pigment extraction residue followed by enzymatically hydrolysis to obtain mainly peptides.

The second multiproduct value chain (MP2) uses *I. galbana* (‘N^+^-biomass’) and aims at extracting pigments as most valuable product. The process consists of:

– High pressure pigment extraction.
– Alkaline extraction of proteins from the pigment extraction residue.

The third multiproduct value chain (MP3) considers *N. gaditana* cultivated under nutrient limitation to enhance the TAG content (‘N^–^-biomass’). The process chain is mainly based on the single product value chain V for oil extraction (see Figure 1). The process consists of the steps:

– Cell disruption by high pressure homogenization.
– Drying.
– Solvent extraction of oil by hexane/isopropanol.
– Alkaline extraction of proteins from the pigment extraction residue followed by enzymatic hydrolysis to obtain mainly peptides.

The fourth multiproduct value chain (MP4) considers again *N. gaditana* that was cultivated under nutrient limitation (‘N^–^-biomass’). This chain first aims at extraction of the lipids, then on pigment extraction, and separation of proteins for peptide production. The process consists of the steps:

– Cell disruption by high pressure homogenization.
– Drying.
– Solvent extraction by hexane/isopropanol.
– High pressure pigment extraction from the defatted biomass.
– Alkaline extraction of proteins from the pigment extraction residue followed by enzymatically hydrolysis to obtain mainly peptides.

### Approach of Process Design

For each value chain, both single and multiproduct, a specific technical process model has been designed and linked to an economic evaluation. All scenario calculations are based on a microalgae production of 10 kton dry weight y^–1^ in a photobioreactor located in the South of Spain at a benchmark level of 2 g L^–1^ biomass concentration. The physico-chemical properties and composition of the biomass was dependent on species and cultivation conditions, (N^+^-biomass and ‘N^–^-biomass’). The biomass composition after cultivation is reported in Supplementary Table S1 on a dry basis (based on duplicate production data). The production data were provided by Fitoplancton Marino S.L. for N^+^-growth conditions (20 ton ha^–1^ year^–1^) and N^–^-growth conditions (12 ton ha^–1^ year^–1^) at a required 500 and 840 ha scale, respectively. To place this area into perspective, it can be compared to the greenhouse horticulture sector in Netherlands with a total area of 9,300 ha in 2016 (Wageningen University and Research, 2017). Cultivation cost estimate were provided by Fitoplancton Marino S.L. based on their experience at production scale. The estimated cultivation costs on 100 ha basis are a projection compared to cost of current 1 ha production scales. Specifically the cultivation cost at 100 ha scale are 4.5 € kg^–1^ (‘N^+^-biomass’) and 7.5 € kg^–1^ (‘N^–^-biomass’). The latter correspond to an N starvation period up to the point where the further lipid accumulation becomes negligible.

The design and calculations for each processing model at industrial scale of 10 kton microalgal dry weight y^–1^ was performed with the aid of the software SuperPro Designer v10.0b3^®^ from Intelligen, Inc^3^. Data on yields, material and energy consumption were based on pre- and pilot size production plants (TRL 5–6). As far as possible, the processes have been modeled in the software adopting continuous operations. In case of batch operations (required for pigment extraction) a detailed schedule has been implemented according to the procedure carried out at lab and pilot-scale as reported in previous literature (Gilbert-Lopez et al., 2015, Gilbert-López et al., 2017a; del Pilar Sánchez-Camargo et al., 2017; Ibáñez et al., 2017) and scaled to the analyzed industrial scale of 10 kton y^–1^ of microalgae biomass. In case of multiple batch operations in sequence, the schedule of each operation has been designed integrated with the other in order to have a complete and coherent procedure for each batch. The presence of tanks for intermediate storage has been not taken into account even in case of batch operations. The complete flowsheets developed in SuperPro Designer are reported in the section with Supplementary Material, including an overview of the inputs and outputs (Supplementary Figures S1–S8). The detailed description of each process step is also given in the Supplementary Material.

### Economic Evaluation

The cost calculations covered capital investment (CAPEX) and operating expenditures (OPEX). Biorefinery capital investments were calculated from the purchase costs of the main equipment (PC) increased by an overall Lang factor of 3.54 (Harrison et al., 2003; Heinzle et al., 2006; Safi et al., 2014). More details on the calculation of the total CAPEX are provided in Supplementary Tables S2, S3. Linear depreciation of the CAPEX over 15 years lifetime with 8% interest rate has been assumed. The working capital is assumed to cover 2 months of OPEX.

Operating expenditures are calculated as the sum of the costs of utilities (electricity, heat, steam, cooling agents), (raw) materials (like solvents), consumables (membranes), wastewater treatment, labor, laboratory costs like quality assurance (Lab/QC/QA), additional facility costs (based on Lang factor), and others (maintenance, operating supplies, overheads, contingencies). Free-on-board costs of raw materials were retrieved from the websites ICIS^4^ and IndexMundi^5^. The costs for utilities and raw materials are specified in Supplementary Table S4. Wastewater treatment costs were assumed constant at 0.45 € m^–3^. For location-dependent costs, Spain was assumed as reference. The costs for CO_2_ adsorption and cultivation are provided and explained in the Supplementary Material. Input of labor was set by considering that an operator can manage up to five continuous process operations simultaneously. In case of batch operations a full-time operator is required per operation. Supervisors and managers were calculated based on three shifts, resulting in a ratio of one manager: three foremen or supervisors: 20 operators (Ruiz et al., 2016). The number of operator workers is calculated on the basis of: (1) the amount of required labor hours per hour of operation; (2) considering three shifts per week. Further information on the assumptions can be found in the Supplementary Material. The expected near-future revenues from both commodity and niche products were determined by the end-users partners of the MIRACLES project team based on their experience and market data (Multi-product Integrated bioRefinery of Algae: from Carbon dioxide and Light Energy to high-value SpecialtiesMulti-product Integrated bioRefinery of Algae: from Carbon dioxide and Light Energy to high-value Specialties, 2017). Niche product market prices are sensitive to an increase of production. The expected near-future product market values were therefore based on a confidential market database developed by the company partner Value for Technology in the project with inputs from the other industrial partners. The assumed near-future foreseeable market prices for each of the products are listed in Supplementary Table S5.

### Sensitivity Analysis

Table 1 shows a summary of the operating conditions as adopted in the process models. A linear local sensitivity analysis has been performed, since large parts of the processes are sequential and the interacting effects of the different operating conditions can be neglected. In particular the effects of the adopted strain is also addressed modulating the operating conditions on the basis of the physico-chemical properties of the whole cell (size and density) at the harvesting operations and of the biomass composition at the extraction operations.

**TABLE 1:** Operating conditions for the biorefinery process steps for benchmark conditions and as used in sensitivity analysis.

**Process step**	**Operating Conditions**	**Benchmark values**	**Range for sensitivity analysis**
Microfiltration	Filtrate flux rate	50	20–100 L m^–2^ h^–1^
	Power input	0.02	0.01–0.05 kW m^–2^
	Cleaning time	4	1–4 h over 24 h
	Concentration factor	10	5–20
	Operating temperature	25°C	n.a.
	Biomass recovery	100%	n.a.
Centrifugation (harvesting)	Minimum limiting particle diameter	^A^1–4 μm	n.a.
	Minimum limiting particle density	1,050 kg m^–3^	1,020–1,100 kg m^–3^
	Sedimentation efficiency	50%	n.a.
	Power to heat dissipation	50%	n.a.
	Operating temperature	25°C	n.a.
	Biomass recovery	98%	n.a.
	Outlet biomass concentration	^#^100–200 kg m^–3^	100–200 kg m^–3^
Spray drying	Final water content	5%	n.a.
	Evaporation rate	30 kg h^–1^ m^–3^	10–50 kg h^–1^ m^–3^
	Air/water ratio	35 kg kg^–1^	n.a.
	Steam/water ratio	1.4 kg kg^–1^	n.a.
	Steam temperature	160°C	n.a.
	Final solids temperature	60°C	50–70°C
	Biomass recovery	100%	n.a.
High pressure homogenization (disruption)	Inlet pressure	^A^600–1,200 bars	600–1,500 bars
	Number of passes	^A^1–2	1
	Power to heat dissipation	100%	n.a.
	Pumping efficiency	70%	n.a.
	Inlet biomass concentration	100 g L^–1^	n.a.
	Disruption efficiency	95%	90–95%
Centrifugation (cell debris separation)	Minimum limiting particle diameter	0.5 μm	n.a.
	Minimum limiting particle density	1,500 kg m^–3^	n.a.
	Sedimentation efficiency	50%	n.a.
	Power to heat dissipation	50%	n.a.
	Operating temperature	25°C	n.a.
	Solids recovery	100%	n.a.
	Solids concentration	300 kg m^–3^	n.a.
	Liquid viscosity	4 cP	n.a.
Ultrafiltration/Diafiltration	Permeate flux rate	30 L m^–2^ h^–1^	10–50 L m^–2^ h^–1^
	Concentration factor	5	n.a.
	Diafiltration volume/liquid volume	2	n.a.
	Filtration time	1 h	n.a.
	Cleaning time	0.5 h	n.a.
	Power input	0.2 kW m^–2^	n.a.
	Operating temperature	25°C	n.a.
	Membrane cut-off	300 kDa	n.a.
Ultrafiltration (protein concentration)	Permeate flux rate	80 L m^–2^ h^–1^	60–100 L m^–2^ h^–1^
	Concentration factor	20	n.a.
	Filtration time	2 h	n.a.
	Cleaning time	0.5 h	n.a.
	Power input	0.2 kW m^–2^	n.a.
	Operating temperature	25°C	n.a.
	Membrane cut-off	8 kDa	n.a.
High pressure extraction	Super critical fluid (SFE) extraction time	^A^1–2 h	n.a.
	Gas expanded liquid (GXL) extraction time	^A^0.3–2.5 h	n.a.
	PLE extraction time	^A^0.5–0.75 h	n.a.
	Biomass loading in the extraction chamber	^A^3.3–10%	18.2–33.3%
Solvent extraction	Temperature	50°C	n.a.
	Time	2–3 h	1–1.5 h
	Solvent to biomass ratio (hexane/ethanol)	15 kg kg^–1^	n.a.
	Solvent to biomass ratio (hexane/isopropanol)	30 kg kg^–1^	n.a.
	Hexane losses in biomass	1 kg kg^–1^	0 kg kg^–1^

*Algal species dependent values are indicated with ^*A*^. ^#^Value chain dependent. For chains that are directly followed by drying 200 kg m^–3^, otherwise 100 kg m^–3^. n.a. means not applicable.*

## Results and Discussion

### Benchmark Performance Single Product Value Chains

**FIGURE 3:**
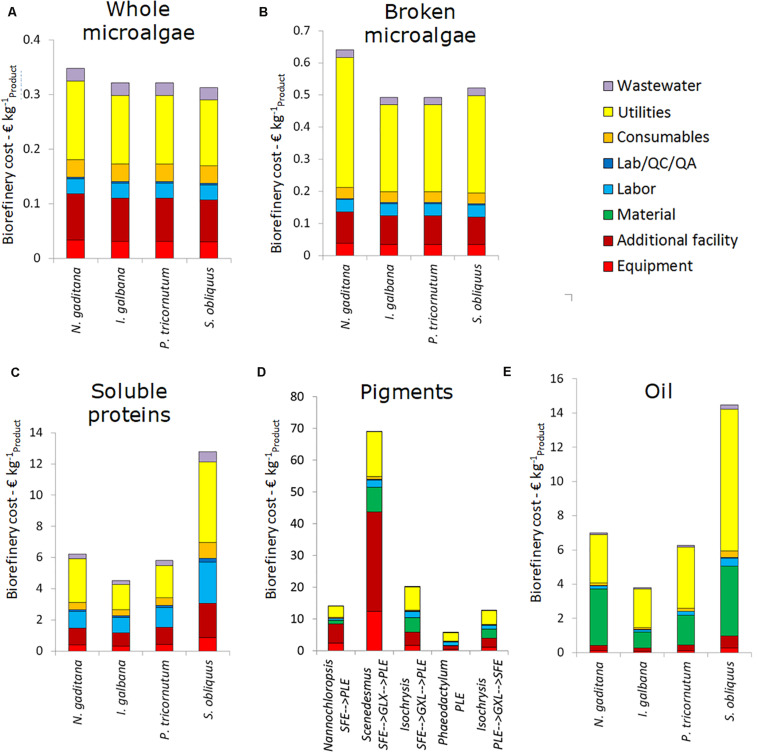
Breakdown of the biorefinery costs (excluding cultivation) for the five single product value chain at 10 ktons y^– 1^ scale. **(A)** Whole microalgae, **(B)** Broken microalgae, **(C)** Soluble proteins, **(D)** Pigments, and **(E)** Oil. Data are expressed as costs per unit of main product. SFE, supercritical fluid extraction; GXL, gas expanded liquid extraction; PLE, pressurized liquid extraction.

For each chain the cultivation costs of 4.50 and 7.50 € kg^–1^ are most of the time the major costs of the value chain. In general, every microalgal chains will benefit from lower cultivation costs. The biorefinery costs (excluding cultivation) per kg product are given in Figure 3 for each of the microalgae strain-product scenarios. The results specify the contribution of the different cost components to the total biorefinery cost.

For whole microalgae, the biorefinery costs (excluding cultivation) are 0.30–0.35 € kg^–1^. The range in costs is caused by the difference in cell diameter between the microalgae species. *S. obliquus* is the cheapest to process as result of the large cell diameter. The cost for utilities account for about 50%, the facility costs (related to equipment facilities, installation, piping, etc.) are another significant cost factor. For whole microalgae the majority of capital expenses is due to the membrane facilities. The steam use in the dryer contributes most to the operating expenses, followed by the electricity use of the membrane filtration.

For broken microalgae the utility costs increase as homogenization is energy intense. This leads to biorefinery costs of 0.50–0.65 € kg^–1^ broken microalgae, with a share for utilities above 50% of the costs. The total costs are thus strongly influenced by the ease of cell disruption. In all cases, the overall yield of broken microalgae on the whole process is around 93%, due to the combination of harvesting losses (∼2%) and losses due to incomplete cell disruption (∼5%).

Processing biomass while aiming for soluble proteins results in biorefinery costs (excluding cultivation) of 4.50–12.80 € kg^–1^ soluble purified protein. The lowest biorefinery costs are for processing *I. galbana* (high soluble protein content and easy to break) and the highest for *S. obliquus* (low protein content and low disruption and separation yield). The large difference in soluble protein yield and, in general, in cell disruption efficiency among the algal strains is also reported in literature (Safi et al., 2014; Günerken et al., 2015; Show et al., 2015).

The overall protein product yields with respect to the initial amount of biomass are for all microalgae species below 10%. As a result, the biorefinery processing costs per kg of product are significantly higher compared to whole and broken microalgae. The utilities contribute significantly to the costs. Furthermore, the relative contribution of labor to the total costs is higher due to the ultrafiltration and diafiltration processes.

The range of pigment processing cost is quite broad: 5.80–68.95 € kg^–1^ pigment product. It mainly depends on the microalgal species that affects the design of the extraction process in terms of capacity and yield. Utility, materials and equipment (related) costs are most relevant in these chains. For the pigment chains all data are expressed per unit of product in which pigments are dissolved. The reason is twofold: (1) pigments are never extracted in pure form, and (2) pigments are frequently unstable and to ensure stability at long term storage they are often dissolved in oil. Drying of the biomass is needed when the first extraction step is supercritical fluid extraction (SFE). The product yields with respect to the initial amount of biomass vary from 9.1% for *S. obliquus* to 24.4% for *N. gaditana*. The steps starting with pressurized liquid extraction (PLE) do not require drying and have product yields around 20%. The capital expenses are mainly due to the investment costs for the extraction vessels. The extraction processes also contribute most to the utility and material costs.

The biorefinery costs for oil are lower than for pigment, i.e., 3.80–14.45 € kg^–1^ extracted oil. The solvents and utilities used for lipid extraction contribute most to these costs. Equipment costs only contribute marginally to the costs. The overall product yields vary between 8.3% for *S. obliquus* and 29.1% for *I. galbana*. *N. gaditana* is also promising for lipid production with an overall yield of lipid extraction of 21.9% of the initial biomass.

### Process Optimization: Effect of Scale and the Most Relevant Operating Conditions

**FIGURE 4:**
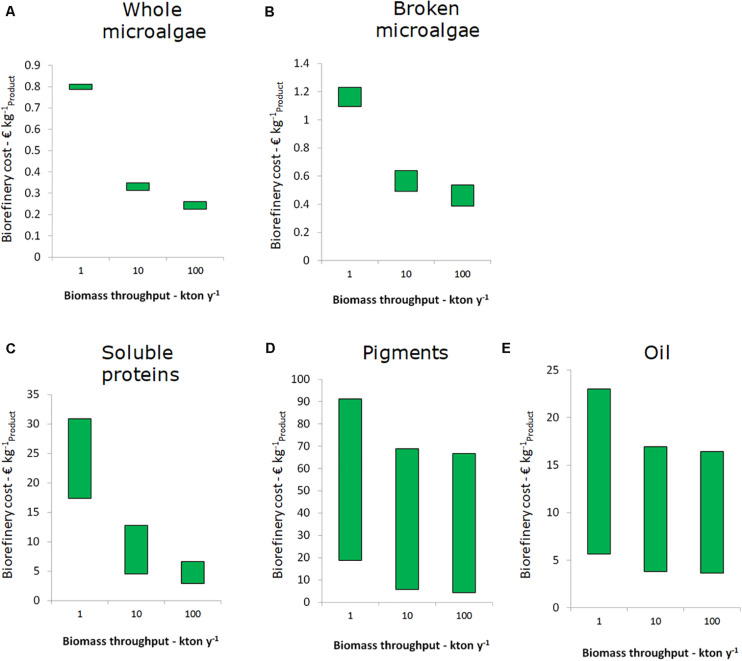
Effect of processing scale on the biorefinery costs (excluding cultivation) for the single product value chain, per unit of main product. **(A)** Whole microalgae, **(B)** Broken microalgae, **(C)** Soluble proteins, **(D)** Pigments, and **(E)** Oil. The bars indicate the range of costs, which is species dependent.

The results above were based on a facility with 10 kton per year capacity. The biorefinery costs for facilities of 1, 10, and 100 kton capacity per year are shown in Figure 4. For all five single product value chains the decrease in biorefinery costs is significant from 1 to 10 kton y^–1^ throughput. For SP value chains I, II, and III cost savings of 50% are feasible. For the other chains the difference in costs is smaller, since their costs are for a large part determined by operating expenses. The cost reduction from 10 to 100 kton y^–1^ is less for all chains and negligible for pigments and lipids. Overall, the results indicate that biorefineries should have a throughput of 10 kton y^–1^ or more to benefit from economy of scale.

**FIGURE 5:**
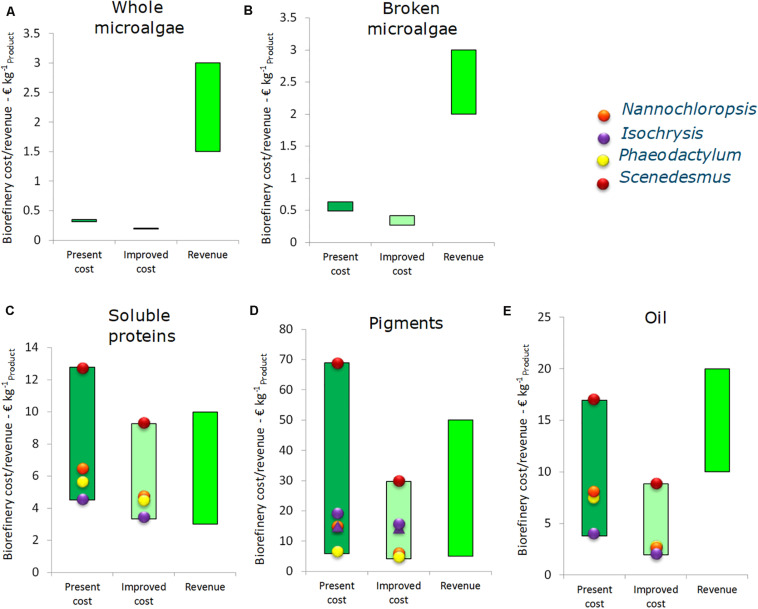
Costs and revenues of the biorefinery process for single product value chain at 10 ktons y^– 1^ scale (excluding cultivation), expressed per unit of main product. **(A)** Whole microalgae, **(B)** Broken microalgae, **(C)** Soluble proteins, **(D)** Pigments, and **(E)** Oil. Costs are reported for benchmark scenario (first bar) and for the scenario leading to reduced cost (second bar, results sensitivity analysis and linear optimization). The revenues (third bar) are further specified in Supplementary Table S5.

A sensitivity analysis combined with process optimization was made for each single product value chain at the 10 kton y^–1^ scale. This was done by optimizing all the adjustable variables according to the ranges reported in Table 1. In Figure 5 the outcomes of the scenarios of benchmark (Section “Benchmark Performance Single Product Value Chains”) and optimized conditions are compared with potential range of revenues from each primary product. Cultivation costs are excluded in this profitability analysis.

For both whole and disrupted microalgae the biorefinery cost are relatively low compared to the potential revenue, as a result of the high product yield and simple structure of harvesting and further biomass processing. By optimizing the operating conditions the biorefinery cost (excluding cultivation) for whole microalgae can be reduced slightly from 0.30–0.35 to 0.20 € kg^–1^ whole microalgae. The potential revenue ranges between 1.5 and 3.0 € kg^–1^. For disrupted microalgae the costs can be lowered from 0.50–0.65 to 0.25–0.40 € kg^–1^ by optimizing the harvesting and disruption conditions. For soluble purified proteins the optimization reduces the biorefinery costs from 4.5–13 to 3.3–9.2 € kg^–1^. This reduction, however, does not necessarily lead to a profitable chain; the revenues for proteins are in the same order of magnitude. However, the value of insoluble components is estimated to be 3.00 € kg^–1^. In the SP protein chain for every kg of soluble protein also 9.68 kg insolubles are produced, thus potentially around 29 € can be gained from these insolubles per kg soluble protein. This is higher than the revenue of the proteins (4.75–8.25 € kg^–1^), thus the main product here is actually the insoluble fraction. For strains characterized by both high processing costs and low yields, such as *Scenedesmus*, the soluble proteins value chain has a small chance to become profitable in this scenario. Consequently, simplification and further exploitation of the other biomass components will increase the potential revenue.

The pigment chain optimization can decrease the biorefinery costs from 5.8–69 to 4.1–30 € kg^–1^ product. Compared to the range of potential revenue of pigments (14–31 € kg^–1^) the effect of the optimization is relevant to achieve a profitable process. In particular, the reverse extraction process applied to *Isochrysis* and the single PLE extraction process applied to *Phaeodactylum* (see Supplementary Material) appear as most suitable candidates for this single product value chain. In the first case, the absence of a drying step reduces the process cost significantly. In the second case, the combination of high yield obtained in a single step process makes the process competitive. However, also in these chains a large amount of biomass remains unused (75–80%) and it does make sense to sell the biomass residue for (aqua)feed worth 0.50 € kg^–1^ residue. This would generate an additional 2.0–5.0 € for each kg of pigment extract.

Optimization indicates that the oil biorefinery costs can be decreased from 3.8–14 to 1.9–8.8 € kg^–1^ oil, which is significantly lower than the potential revenue of the extracted oil (10–29 € kg^–1^ of oil). Again, the biorefinery process based on *Scenedesmus* shows the most critical situation due to the low extraction yield (8%). The exhausted biomass has, however, a residual value for feed or material applications and is potentially worth up to 2.00 € kg^–1^ residue, leading to 4.8 € additional revenue per kg oil extract. *Isochrysis* and *Nannochloropsis* are the most likely candidates for such a type of biorefinery, due to the high yield (29 and 22%). These yields are achieved with biomass cultivated under nitrogen replete conditions. Microalgal biomass obtained by growth under N-limitation or N-starvation can contain up to 50–65% lipids (Breuer et al., 2012). Especially *Nannochloropsis* is interesting due to the lipid profile (Draaisma et al., 2013). Thus, a suitable strategy could be to produce *Nannochloropsis* biomass under N-limited conditions and to exploit the half of the unused biomass.

### Multiproduct Value Chains

**FIGURE 6:**
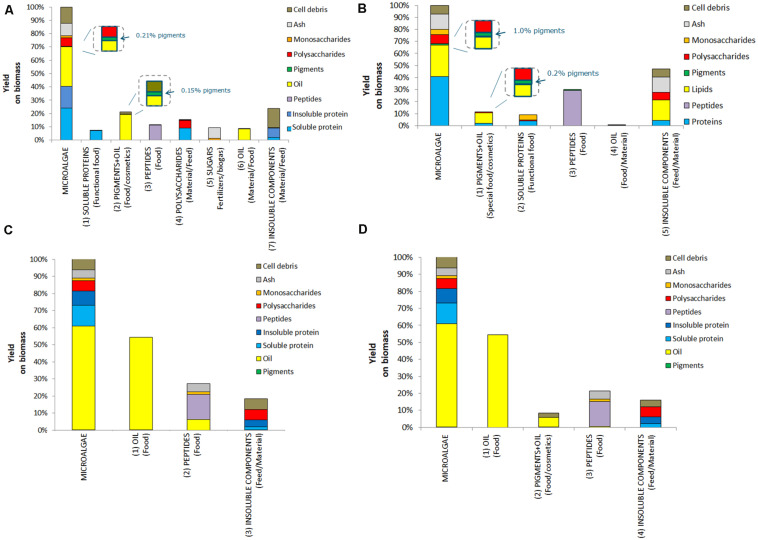
Initial biomass composition and detailed breakdown of the composition of the product streams for multiproduct value chains (MP); **(A)** MP1, **(B)** MP2, **(C)** MP3, and **(D)** MP4.

**FIGURE 7:**
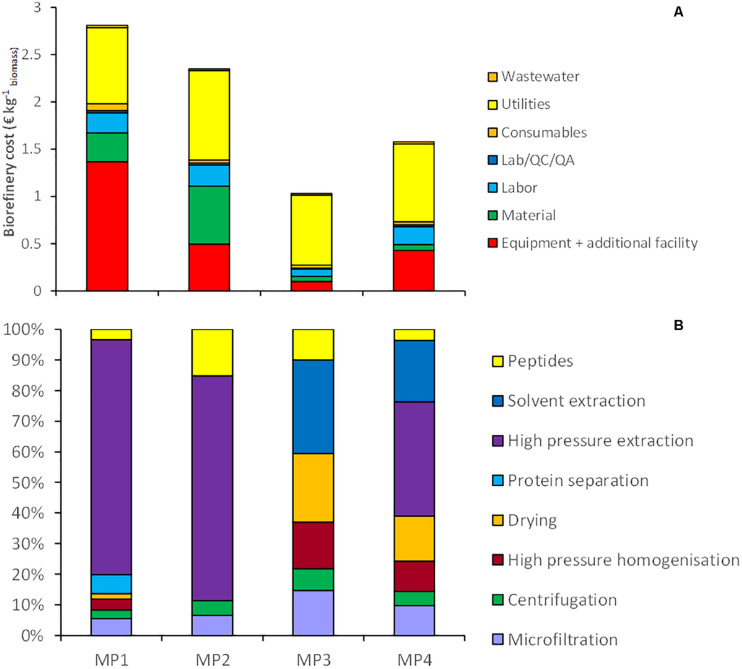
**(A)** Breakdown of the biorefinery costs (excluding cultivation) for the multiproduct value (MP) chains at 10 ktons y^– 1^ scale, expressed per unit of initial biomass, **(B)** contribution of each biorefinery step to total biorefinery costs. Protein separation = Centrifugation + UF/DF + UF; Peptides = alkaline extraction + enzymatic hydrolysis.

**FIGURE 8:**
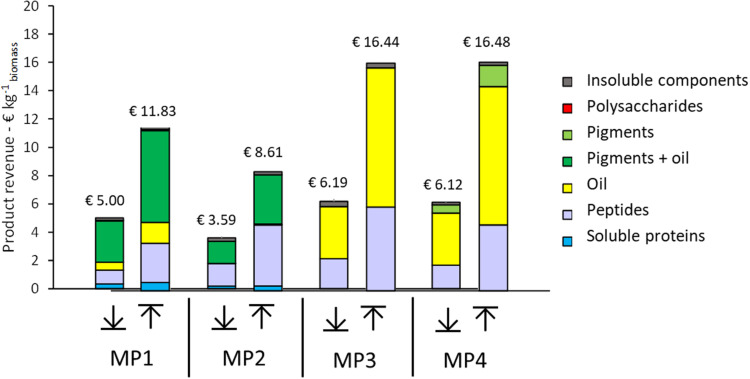
Range of potential product revenue expressed per unit of initial biomass for each multiproduct (MP) value chain. (↓) worst case revenue estimates, (↑) best case revenue estimates.

Four multiproduct value chains have been selected based on the results of the single product value chains. The first multiproduct value chain MP1 was designed for *N. gaditana*. Although the single value chain III with *Isochrysis* resulted in slightly higher product yields and lower costs, *Nannochloropsis* was preferred due to its potential product range and opportunities to diversify the multiproduct chains. Figure 6A shows the distribution of the microalgal components over the various products for MP1. The potential of enhanced biomass exploitation is clearly shown. In comparison to single product value chain III the biomass exploitation in the form of marketable products with potential increases significantly from 7 to 97%. As expected, the biorefinery costs (without cultivation) increased to 2.8 € kg^–1^ biomass for multiproduct value chain MP1 (Figure 7). However, the broader product portfolio also increased the overall revenue to 5.0–12 € kg^–1^ biomass (Figure 8). The breakdown of the overall biorefinery costs is shown in Figure 7A. Equipment and utilities are the main cost contributors. Figure 7B illustrates the contribution of the biorefinery steps, the high pressure extraction is the most costly biorefinery step followed by solvent extraction. The increase of biorefinery costs due to the alkaline extraction and enzymatic hydrolysis is low.

The second multiproduct value chain MP2 (Figure 2B) can be compared to the single product value chain IV for pigment extraction. Based on the costs and revenue analysis of the single product chains the reverse extraction process has been chosen as the most suitable technology for extracting the pigments. *I. galbana* appeared the most suitable strain for this process since no cell disruption was needed before the extraction, the high product yield, and low costs compared to the revenue. Figure 6B shows the microalgal components in each product for multiproduct value chain MP2. Again the biomass usage potentially increased from an initial 11% (single value chain) to 98%. In this multiproduct chain the biorefinery costs increase slightly to 2.3 € kg^–1^ biomass, with a potential revenue ranging from 3.6 to 8.6 € kg^–1^ biomass (Figure 8). In this chain the utilities are the main cost contributor, followed by material use and equipment. This is again due to the high pressure required for pigment extraction, which, although a costly process, also generates a major share of the revenue (Figure 7B).

The third multiproduct value chain MP3 (Figure 2C) is mainly based on single product value chain V and considers using N^–^-biomass of *N. gaditana* which contains more TAG-rich oil than N^–^-grown biomass. According to the costs and revenues of the single product value chain the extraction with a mixture of hexane/isopropanol (3:2) applied to dry biomass has been selected as the most favorable approach. Figure 6C shows the overall microalgal components starting with the N^–^-biomass and a breakdown of the composition and the extracted components. With the multiproduct cascading the biomass use increased from 33% till almost 98%. The biorefinery costs are 1.03 € kg^–1^ biomass, the lowest of all multiproduct chains (Figure 7A). Large part of the cost is due to the utilities (Figure 7A), whose consumption is quite similar in all the process steps (Figure 7B). The revenues range between 6.2 and 16.4 € kg^–1^ biomass (Figure 8). Around 60% of the revenue is due to the oil and about 35% due to the peptides product. The fourth multiproduct value chain MP4 also considers N^–^-biomass of *N. gaditana* In addition to MP3, here also pigments are extracted. Figure 6D shows the overall composition of the N^–^-biomass and a breakdown of the extracted components. In the single product chain only one third of the biomass was exploited. The biorefinery costs (excluding cultivation) are 1.55 € kg^–1^ biomass (Figure 7A). The revenue ranges between 6.1–16.5 € kg^–1^ biomass (Figure 8) and is thus similar to that of the MP3 chain, despite the additional extraction of pigments (8% of product mass). In MP4 the peptide fraction decreases from 27% (MP3) to 21% (MP4). In this chain the remaining insoluble fraction is 18% of the starting biomass (Figure 6D), but with a lower value than in MP3 due to the extracted oil and pigments.

### Discussion: Profitability Analysis

**FIGURE 9:**
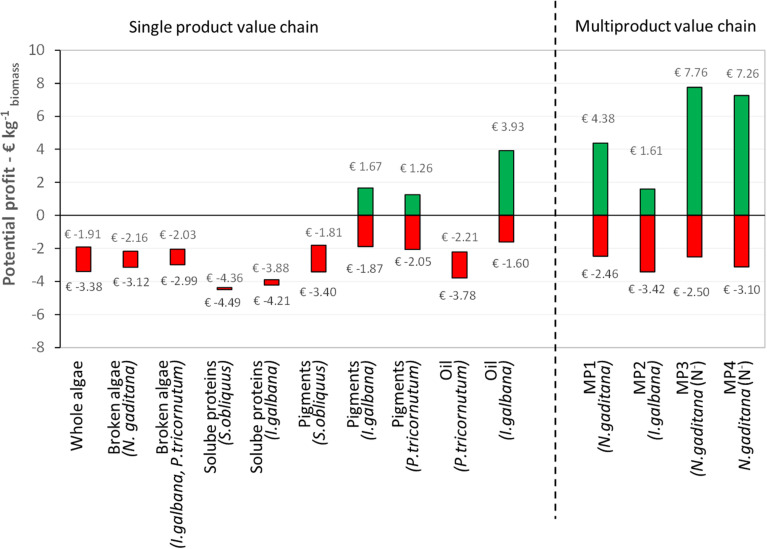
Potential profit expressed per unit of initial biomass for the single and multiproduct value chains at 10 ktons y^– 1^ scale, based on the total production costs (including CO_2_ capture, cultivation, and biorefinery) and the potential revenues. MP, multiproduct value chain.

The potential profit ranges of the five single product and four multiproduct value chains at 10 kton y^–1^ scale are shown in Figure 9. For each single product value chain, the data for the strains with the highest and lowest costs are shown. The profit range was calculated based on the revenues (worst and best case estimates) and cost (including CO_2_ capture and cultivation). The center points of the profit ranges are used to evaluate the profitability of the value chains. A positive center point indicates that the estimated revenue is sufficient to balance the costs, a negative center point suggests that the estimated revenue is unlikely to be sufficient to balance the cost. No cash flow analysis was performed. Below the results are discussed together with other proposed value chains in literature.

The results show that for each chain the cultivation costs (4.50 € kg^–1^ for N^+^-biomass and 7.50 € kg^–1^ for N^–^-biomass) are most of the time the major production costs. One should realize that the used estimates of cultivation costs are already a projection compared to the current 1 ha production. In general, all SP and MP chains will benefit from lower cultivation costs, as also indicated previously (Laurens et al., 2017). The benchmark size of the production facility reported here as well as in literature (100 ha; Beal et al., 2015; Ruiz et al., 2016) can affect the market equilibrium, maybe ending in the demand saturation for some high value products.

The scenarios for whole and broken microalgae, as well as for soluble proteins (including the residue as co-product) always lead to a negative potential profit at 10 kton y^–1^ scale. This means that the combination of cultivation and biorefinery costs are higher than the generated revenues. The soluble protein value chain is also least investigated in literature in terms of process design and economic evaluation. Asiedu et al. (2018) provided a TEA for production of a dry protein hydrolysate powder (amino-acid and peptides) produced via flash hydrolysis from *S. obliquus* cultivated in open ponds. Assuming 81% of protein extraction and conversion, they ended with production cost of 2.99 € kg^–1^ protein and a minimum selling price of 4.31 € kg^–1^ protein (Asiedu et al., 2018).

For pigments and oil, the profit of the process depends on: (I) the oil and pigments content of the microalgal strain; (II) the efficiency of high pressure extraction; (III) the selling price of the pigments or oil. These figures are also confirmed in literature. Quinn and Davis (2015) have reviewed more than 20 techno-economic analysis for biodiesel production, which is a single product chain based on oil. In that work they showed that the large variety in costs (0.27–9.7 € kg^–1^ biofuel) is mainly related to the cultivation technology (open pond vs. closed photobioreactor) and on the lipid yield from cultivation (Quinn and Davis, 2015). Thomassen et al. (2016) have analyzed several scenarios of beta-carotene and astaxanthin production, confirming that the pigment content and extraction efficiency mainly affect the process profitability (Thomassen et al., 2016). In contrast to our work (range of 13.75–31.25 € kg^–1^ pigment), they consider a small market for their product and assume higher pigment selling prices, i.e., for beta-carotene 1180 € kg^–1^ and for astaxanthin 5113 € kg^–1^.

For the multiproduct chains the profit range increases, with a positive center point (average) for MP1, 3, and 4. For these chains the worst-case estimate of revenue is thus not sufficient to balance the cultivation and biorefinery costs. For MP1 and MP2, large part of the revenue comes from pigments and peptides, while for MP3 and MP4 significant revenues are obtained from the oil and peptide products (Figure 8). Therefore, the efficiencies of the process steps related to those pigment, peptide and oil extraction and purification are the main factors affecting the process profitability. In contrast at this stage, the contribution of the additional co-products such as soluble proteins to the whole process economy seems not relevant. Additional insights in the product functionality, quality and their market size are needed to narrow down the wide range of foreseen product revenues and resulting profits.

To develop a profitable micro-algae production chain a combination is required of (1) technological innovations enabling cost reductions, especially in micro-algae production, (2) developing multiproduct biorefinery concepts aimed at valorizing the full biomass through the cascading principle, and (3) deriving a range of new specialty products with applications in food, aquaculture and non-food. Cascading increases the biorefinery costs significantly, but this is compensated by the enhanced overall biomass revenue, provided that the sequence of operations does not affect the yield and properties of the final products. When these conditions are met the achieved overall biomass revenues of the integrated, multiproduct chains enable an economically competitive microalgae biorefinery.

## Data Availability Statement

The raw data supporting the conclusions of this article will be made available by the authors, without undue reservation.

## Author Contributions

PS and GO performed TE evaluation and interpretation, drafted the manuscript, and took the responsibility for the integrity of the work as a whole, i.e., from conception until the finished article. PS coordinated data acquisition. GO developed and optimized the flow sheets. PS, GO, EB, LS, and JR conceived and designed the study. ME, RW, and JR obtained funding. All authors contributed to the selection of the single value chains, have critically reviewed the proposed multiproduct chains and results, and have critically revised the manuscript, read, and approved the submitted version.

## Conflict of Interest

The authors declare that the research was conducted in the absence of any commercial or financial relationships that could be construed as a potential conflict of interest.

## Footnotes

1 http://www.iea-bioenergy.task42-biorefineries.com/en/ieabiorefinery/Activities-1.htm

2 The species has recently been renamed to *Acutodesmus obliquus*, but in the remainder of this work *Scenedesmus obliquus* is used.

3 www.intelligen.com

4 www.icis.com

5 www.indexmundi.com
