# Supplementary material for: Design of Value Chains for Microalgal Biorefinery at Industrial Scale: Process Integration and Techno-Economic Analysis
Source: Front Bioeng Biotechnol. 2020 Sep 8;8:550758. doi: 10.3389/fbioe.2020.550758 (PMC7510460; doi:10.3389/fbioe.2020.550758)
Supplement: Supplementary file 1 [file Data_Sheet_1.docx]

Supplementary Materials

This supplementary materials contains the detailed description of each process step as well as the complete flowsheets developed in SuperPro Designer®, including an overview of the inputs and outputs. The procedure for calculating the total capital investment, CAPEX and OPEX are further specified. Prices for utilities, raw materials and product revenues are provided.

# Detailed description of each process step

# CO_2_ adsorption

The MIRACLES project uses air capture and concentration of CO_2_ gas from ambient air to enhance microalgae cultivation (Brilman et al., 2013). Specific solid sorbents are used to first capture CO_2_, which is released into a sweep gas during sorbent regeneration. Water vapour, temperature increase, pressure reduction and air as sweep gas can all be used for sorbent regeneration. The novel system is based on a radial flow reactor for CO_2_ adsorption and a fluidized bed for desorption using air purge. The solid sorbent circulates between the adsorber and desorber. The advantages of solid sorbents are the lower specific heat compared to aqueous sorbents and desorption can take place at moderate temperatures (in this case at 56 ºC). The obtained CO_2_ enriched air is used directly in algae cultivation. On average 20,000 ton CO_2_ is required yearly for cultivation. This is bound from 4.4·10^10^ m^3^ of air using 1·10^5^ kg sorbent at 50% capture efficiency and 400 ppm of CO_2_ in air. A low pressure drop contactor is used to minimize the energy for air-sorbent contacting (Yu and Brilman, 2017). The total CAPEX and OPEX were calculated to be €0.088 kg^‑1^ CO_2_ , including equipment cost, utilities, material use and labour (Yu, 2018). The majority of these cost are OPEX.

# Cultivation

All benchmark calculations are based on 10 kton dry weight y^–1^ algae production in a tubular photobioreactor at 2 g L^–1^ biomass concentration. The cultivation strategy was chain dependent and two cultivation conditions have been addressed in order to optimise the content of the main product to be extracted and purified: optimum nutrient replete conditions (N^+^-biomass) and nutrient limitation by nitrogen starvation to enhance the triacylglyceride content (N^-^-biomass). The data were based on actual produced biomass from the facilities of Fitoplancton Marino s.l. in the South of Spain. The projected production costs for the scale of annually producing 10 kton algae are €4.50 kg^‑1^ algae (N^+^-biomass) and €7.50 kg^-1^ algae (N^-^-biomass). These costs comprise the required CAPEX and OPEX, including utilities, material, labor and maintenance. The projection is based on industrial experience of the company about costs of the facilities and of the needed labour. The costs of the chemicals as well as the utilities are assumed not to change with the production scale. The higher cultivation costs for lipid enriched biomass are due to the extended cultivation period and the corresponding higher use of utilities and materials.

# Microfiltration

For harvesting a combination of tangential flow microfiltration (MF) and centrifugation was selected, a combination that minimizes the electricity consumption and overall costs of the harvesting process (Baerdemaeker et al., 2013). Tangential flow microfiltration, also referred to as a submerged membrane reactor, is used as primary step of biomass concentration in all scenarios. The advantage of this technique is that the permeate can be recycled to cultivation (MIRACLES (Multi-product Integrated bioRefinery of Algae: from Carbon dioxide and Light Energy to high-value Specialties), 2017) (Bastiaens et al., 2017). The main operating conditions for MF are: biomass concentration factor (CF), biomass retention factor, permeate flux rate, and specific electricity consumption. The CF ranged between 5–20 which is corresponding to a maximum level of final biomass content of 4%_DW_ (Gerardo et al., 2015). A retention factor of 100% has been assumed (Rossignol et al., 1999; Bilad et al., 2012; Show et al., 2013; Gerardo et al., 2014a; b; Gerardo et al., 2015). Generally, permeate flux rate range between 10 and 100 L m^–2^ h^–1^. In literature also values close to 150 L m^–2^ h^–1^ can be found (Rossignol et al., 1999; Danquah et al., 2009; Baerdemaeker et al., 2013). However, in the range of 50–100 L m^–2^ h^–1^ a critical flux is established at which membrane fouling can happen (Baerdemaeker et al., 2013). Backflushing is the common procedure to clean the membrane periodically. In the process models 85% of filtration time and 15% of backflushing for cleaning has been assumed. Electricity consumption strongly depends on the permeate flux rate and the transmembrane pressure. The electricity consumption is expressed as the electricity per unit of membrane area (kWh m^–2^) or per unit of permeate volume (kWh m^–3^). The first choice has been adopted in the process models. In our analysis the power input ranged between 0.01 and 0.05 kW m^–2^ , which corresponds to 0.5–1 kWh m^–3^ as reported in literature (Danquah et al., 2009; Gerardo et al., 2014b). The maximum area of the membrane module has been fixed at 150 m^2^. Energy balance have been set by assuming 100% of heat dispersion of the power input.

# Centrifugation (harvesting)

Centrifugation is used as second concentration step. A microalgae concentration of 20%_DW_ is assumed to leave the centrifuge. At this concentration the algal stream shows a plastic behaviour. Therefore, the final biomass concentration after centrifugation was reduced to 10%_DW_ for the chains that require cell disruption by homogenisation.

Disk–stack centrifuges are the most suitable for microalgae harvesting as large values of centrifugal acceleration are required due to the low cell density and size. Disk–stack centrifuges have been characterised by their volumetric throughput (m^3^ h^–1^) and equivalent sigma factor (Σ – m^2^). It has to be underlined that the equivalent sigma factor (Σ – m^2^) depends on the square of the rotation speed of the centrifuge, meaning that a high value of Σ involves a high electricity consumption. The largest disk–stack centrifuges on the market can treat up to 90 m^3^ h^–1^ and are characterized by Σ values of 220,000 m^2^ (Gerardo et al., 2015). In practice, the required centrifuge capacity and size are selected on the basis of the strain properties and desired recovery according to the procedure reported in (Coons et al., 2014).

A critical parameter affecting the size of the centrifuge is the cell density and diameter. Among the strains adopted in this project the cell density has been assumed to be 1050 kg m^–3^. The size of the cells depends on the strain: *Nannochloropsis* 2–3 µm, *Isochrysis* and *Phaeodactylum* 3–5 µm and *Scenedesmus* 5–7 µm. To determine the critical value for optimum recovery the centrifuge has been designed with the lowest cell sizes, i.e. 1 µm for *Nannochloropsis*, 2 µm for *Isochrysis* and *Phaeodactylum* and 4 µm for *Scenedesmus*. The typical electricity consumption is calculated automatically in SuperPro Designer and ranges between 1.0–1.4 kWh m^–3^.

# Drying

Dry biomass is required in single product value chains I and II to obtain dried product and in single product value chains IV, V and multiproduct chains 1,3 and 4 for efficient extraction. On lab-scale freeze drying is frequently applied. However, this is a costly and laborious process at larger scale. On industrial scale spray drying is the most efficient drying technique. Spray drying can treat algae paste at 20%_DW_ and has as advantage that the product–steam contact time is very short. Medium pressure steam is used to warm the air up to an inlet temperature of 160 °C, while the outgoing temperature of the dry biomass is set to 60 °C. According to this the amount of 5 atm steam and of air per unit of evaporated water were set to 1.4 and 35 g per kg­ of biomass, respectively. The residual water content after drying was fixed at 5%_DW_. The maximum industrial scale of spray dryers was set to 500 m^3^ of volume. The heat consumption can be calculated from the steam demand assuming 2100 kJ kg^‑1^ steam as latent heat. Electricity consumption consumed for the spray injection was set to 0.02 kWh kg^‑1^ feed, which corresponds to a system using rotary wheel atomizers and injection pressure between 20 and 100 bars (Mujumdar, 2014).

# Cell disruption

High pressure homogenisation (HPH) has been adopted for cell disruption. The degree of disruption is influenced by the pressure and the number of passes. The disruption degree is ranging between 76–99% (Günerken et al., 2015). *Nannochloropsis* has the most tough cell wall: at a pressure of 1200 bar and one pass about 85% of the cells are disrupted. Two passes can lead up to 98% of disruption. Similar degrees of disruption have been found with one pass for *Scenedesmus* and with half of the inlet pressure for *Phaeodactylum* and *Isochrysis*. The electricity consumption for pumping depends on the number of passes and inlet pressure. It ranges between 24 and 95 kWh m^–3^, assuming a pumping efficiency of 70%. In literature larger values can be also found for *Nannochloropsis* (70–250 kWh m^–3^), with more than 90% of disruption (Günerken et al., 2015). The maximum capacity of a homogeniser has been set at 5 m^3^ h^–1^, according to data courteously provided by GEA.

# Centrifuge (cell debris/liquid separation)

In single product chain III and multi–product chain 1 a second centrifugation step is required. This centrifugation step is used to separate the cell debris from the liquid phase containing soluble proteins and sugars. As such membrane fouling is avoided at the subsequent ultrafiltration step. This centrifuge demands more electricity per treated volume than a harvesting centrifuge. The ingoing stream contains whole algae, broken algae and the water–soluble intracellular components (released during cell disruption). Coherently it has been assumed that the whole algal cells and cell debris (containing insoluble proteins, membrane lipids and the other mix of components) transfer during centrifugation to the solid phase. According to that, the critical size and density of cell debris to be separated have been set to 0.5 µ m and 1500 kg m^‑3^ respectively. The liquid viscosity has been set at 4 cP according to estimates from literature for algae mixture at 10%_DW_ (Wileman et al., 2012). The electricity consumption is calculated according to the same procedure reported for harvesting.

Tests performed at lab scale revealed that to have a high efficiency of separation of the supernatant from the pellet more than one centrifugation step was required. This is due to the high cell density adopted during the HPH disruption. The biomass and protein yields collected after three washing and centrifugation steps are reported in the supplementary materials (Figure S 10A–B). The yields are based on the mass collected from the various supernatant fractions as well as the leftover in the remaining pellet after the third centrifugation step. The results show that the yield of proteins in the supernatant increased significantly for almost all strains: 9%🡪19% for *Scenedesmus*; 32%🡪46% for *Nannochloropsis*; 18%🡪59% for *Phaeodactylum* and 29%🡪66% for *Isochrysis*. Therefore, three washing–centrifugation steps were applied except for *Nannochloropsis*, where two steps are applied since the last step did not significantly improve the final yield.

# Ultrafiltration/Diafiltration

A cascade of two ultrafiltration (UF) steps is used in single product value chain III to first fractionate polysaccharides from water soluble proteins and then to concentrate the proteins (Marcati et al., 2014; Safi et al., 2014; Safi et al., 2017). The first step (hydrophilic membrane of 300 kDa) can retain polysaccharides like starch as well as lipid molecules such as pigments. The second step (hydrophilic membrane of 8 kDa) retains water soluble proteins. According to experiments (Safi et al., 2017) a batch–wise UF test with an inlet concentration of 100 g L^–1^ gives a maximum concentration factor for starch of 9.8 at 300 kDa and a maximum protein recovery of nearly 90% in the permeate. The permeate flux rate can range between 10 and 50 L m^–2^ h^–1^. The UF is stopped when 80% of the volume is filtered. The second UF step is required for concentrating proteins in the retentate. This UF is operated as a diafiltration (DF) step with two additional dia–volumes of buffer solution The maximum concentration is set at 100 g L^–1^, even if no experimental observation has indicated a clear gel limit. Tests carried out within the MIRACLES project indicate that the permeate flux rate can be constant at 80 L m^–2^ h^–1^.

The power input is estimated to be in the order of 0.2 kW m^–2^, considering both the permeation and recirculation between the tank and the membrane, resulting in 4–20 kWh m^–3^ of permeate.

# High–pressure extraction procedure

In single product chain IV and multi–product chain MP2 a high pressure extraction is performed to sequentially extract pigments, valuable lipids and soluble proteins. The procedures consists of sequential steps including Supercritical Fluid Extraction with CO_2_ (SFE), Gas eXpanded Liquid extraction with CO2 + ethanol (GXL) and Pressurized Liquid Extraction with subcritical Water (PLE) (Gilbert-Lopez et al., 2015; Gilbert-López et al., 2017a). The procedure can be arranged in several ways, depending on the strain and the water content of the biomass: direct, employed when extracting dry biomass (SFE🡪GXL🡪PLE, or SFE🡪PLE), and reverse, used for the extraction of wet biomass (PLE🡪GXL🡪SFE), or single step (SFE or GXL or PLE) as reported elsewhere (Gilbert-Lopez et al., 2015; del Pilar Sánchez-Camargo et al., 2017; Gilbert-López et al., 2017a; Gilbert-López et al., 2017b; Ibáñez et al., 2017). In the direct procedure, the first step aims at extracting non polar lipids and pigments. GXL is performed for extracting polar–lipids and residual part of pigments. The use of subcritical water as PLE is reported in case of extraction of hydrophilic components such as proteins and sugars. In the reverse procedure the schedule of the operation is reversed with few modifications. A detailed schedule with conditions for each operation of the procedure is reported below. The process includes pre–treatment steps such as cell disruption and drying, depending on the strain used. For example, cell disruption is required to improve the yield of extraction when the strain has tough cell walls. The main advantage of the reverse procedure is the possibility to start the sequence with wet biomass, thereby skipping the drying step.

Solvents separation and recycling is carried out by a sequence of flash, condensation, pumping and heating again at the extraction conditions. CO_2_ losses of 5% are taken into account, as well as ethanol losses which are depending on the temperature in the last flash after the GXL extraction step. The overall procedure time ranges between 5 and 8 h. The volume chamber is filled with dry micro–algal biomass at different volume fraction: 3.3% in case of *Scenedesmus* and *Nannochloropsis*, 9.1% in case of *Phaeodactylum* and 33.3% in case of *Isochrysis*. It is expected that at industrial scale several extraction vessels must be operated in parallel since the overall industrial vessel size can range between 0.01 and 10 m^3^ (Rosa and Meireles, 2005; 2009; Rocha-Uribe et al., 2014). Electricity and heating/cooling agent demands are automatically calculated from the energy balance at each operation of the extraction (pumping, flash and cooling) according to the operating conditions as reported in supplementary materials. The cost–model for the pressurised extraction equipment has been derived using literature data (Rosa and Meireles, 2009; Prado et al., 2010; Prado et al., 2012; Coons et al., 2014; Rocha-Uribe et al., 2014; Cavalcanti et al., 2016).

DIRECT PROCEDURE SFE🡪GXL🡪PLE

1) Biomass loading – 5 min

2) Heating at 50°C, Flushing CO_2_ at 1 atm, 2 min

3) Static pressurization at 350 bars – 5 min

4) **SFE – Continuous extraction** by feeding ScCO_2_ at 350 bars, 50°C, 0.023 min^–1^ – 2 h for *S.* *obliquus*, 1 h for *I. galbana*

5) Static depressurization (by venting) at 70 bar – 15 min

6) **GXL – Continuous extraction** by feeding CO_2_ (0.023 min^–1^) and Ethanol (0.0115 min–^1^) at 70 bars 50 °C, – 2.5 h for *S. obliquus*, 2 h for *I. galbana*

7) Flushing with CO_2_ at 70 bars, 50°C, 20 min

8) Static pressurization with water at 100 bars – 8 min

9) **PLE – Static extraction** at 50°C – 0.75 h for *S. obliquus*, 0.5 h for *I. galbana*

10) Static depressurization (by venting) at 1 bar – 3 min

11) Flushing with CO_2_ 0.023 min^–1^ to remove water and dry – 60 min

REVERSE PROCEDURE PLE🡪GXL🡪SFE, only for *I. galbana*

1) Biomass loading (29%w/w) – 5 min

2) Start to keep at 30°C – 5 min

3) Static pressurization with water at 100 bars – 8 min

4) PLE – Static extraction at 30°C – 30 min

5) Static depressurization (by venting) at 1 bars – 3 min

6) Flushing with N_2_ at 10 bars and 30°C to dry water – 40 min

7) **GXL –** **Continuous extraction** by feeding CO_2_ (0.023 min^–1^) and ethanol (0.023 min^–1^) at 70 bars, 50 °C – 1.5 h for *I. galbana*

8) Flushing with N_2_ at 10 bars and 30°C to dry water – 30 min

9) Static pressurization and heating at 300 bars and 70°C with CO_2_ – 30 min (5 min to increase pressure, 30 min to increase temperature)

10) **SFE –** **Continuous extraction** by feeding ScCO_2_ at 300 bars, 70°C, 90min

11) Depressurization (by venting) at 1 bar – 15 min

# Solvent extraction

Solvent extraction is a benchmark technology for lipid extraction. Commonly, hexane is used to extract non–polar lipids from oleaginous crushed biomass such as soybean seeds. In case of microalgae the extraction yield can be improved by adding a polar solvent such as ethanol, butanol or isopropanol to extract also polar lipids (Halim et al., 2012; Halim et al., 2014). The extraction takes place at 40–50°C for 1–3 h, depending on the strain. The extraction efficiency goes up to 0.8–1.0 kg kg^–1^ ingoing oil. Hexane is recovered in an evaporator. A recovery of 95% is common. The heat consumption is calculated from the evaporation heat of hexane. A 10% additional electricity consumption is included.

# Alkaline extraction and enzymatic hydrolysis

The residue from lipid and pigment extraction by either SFE or solvent extraction consists of mainly insoluble proteins. In the multi–product value chains MP1,2,3 and 4 this residue is further processed for extracting and hydrolysing these leftover proteins. The process includes 3 steps: alkaline extraction, pH adjustment and enzymatic hydrolysis. In the alkaline extraction 10% (w/v) microalgae are soaked in 0.1 M NaOH and mixed for 2 h at room temperature, after which the supernatant is collected by centrifugation (>6000g). In the second step the pH of the supernatant is adjusted to pH 6.5 using HCl. In the enzymatic hydrolysis proteases (0.1% (w/v)) are added from a stock and incubated for 1 h at 50 ^o^C. The reaction is terminated by incubation at 90^o^C for 30 minutes. This results in inactivation of the enzyme and pasteurization of the sample. From the hydrolysis a mixture of peptides is obtained with interesting antimicrobial properties for potential use as a food preservative.

# Complete flowsheets developed in SuperPro Designer


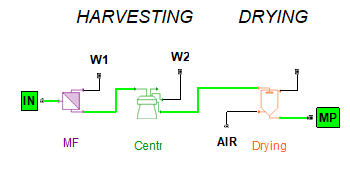


Figure S 1 – SuperPro flowsheet of biorefinery process for single product value chain I (whole microalgae). IN – inlet stream; MP – main product stream; W1,W2 – waste stream . Green line highlights the streams containing the main product

Figure S 2 – SuperPro flowsheet of biorefinery process for single product value chain II (broken microalgae). IN – inlet stream; MP – main product stream; W1, W2 – waste streams. Green line highlights the streams containing the main product.

Figure S 3 – SuperPro flowsheet of biorefinery process for single product value chain III (soluble proteins). IN – inlet stream; MP – main product stream; R1, R2, R3 – residue containing streams; W1, W2 – waste streams. Green and red lines highlight the streams containing the main product and the residues, respectively

Figure S 4 – SuperPro flowsheet of biorefinery process for single product value chain IV (pigments). The pressurised extraction takes into account the most complete procedure involving SFE, GXL and PLE. The flowsheet is simplified when one of them was skipped (microalgae species dependent). HPH and drying are not required for all the strains either. MP – main product stream; R1, R2, R3 – residue containing stream; W1, W2 – waste streams. Green and red lines highlight the streams containing the main product and the residues, respectively.

Figure S 5 – SuperPro flowsheet of biorefinery process for single product value chain V (oil). Solvent extraction can be made either with a mixture of hexane and ethanol (3:2) or with a mix of hexane and isopropanol (3:2), depending on the strain. Green and red lines highlight the streams containing the main product and the residues, respectively.

Figure S 6 – SuperPro flowsheet of the biorefinery process for *Nannochloropsis* (N+)–based multiproduct value chain 1. P1, P2, P3, P4, P5, P6 and P7 are product containing streams. Green lines highlight the streams containing the products.

Figure S 7 – SuperPro flowsheet of the biorefinery process for *Isochrysis*–based multiproduct value chain 2. P1, P2, P3, P4, P5 are the product containing streams. Green lines highlight the streams containing the products.

Figure S 8 - SuperPro flowsheet of the biorefinery process for *Nannochloropsis* (N^-^)–based multiproduct value chain 3. P1, P2, P3, P4 are the product containing stream. Green lines highlight the streams containing the products.

Figure S 9 – SuperPro flowsheet of the biorefinery process for *Nannochloropsis* (N^-^)–based multiproduct value chain 4. P1, P2, P3, P4 are the product containing stream. Green lines highlight the streams containing the products.

Figure S 10 – Yields of biomass (A) and proteins (B) in the supernatants (Surn1, Surn2 and Surn3) that are collected after three washing and centrifugation steps of the disrupted cells and in the leftover pellet after the third washing and centrifugation step (Pellet 3). Data refers to the mass amount recovered in each phase with respect to the initial amount present in the biomass before cell disruption.

# Techno-economic approach and assumptions

For each strain – value chain scenario combination the following were analysed:

1. Mass balances, derived from experimental data generated in the MIRACLES project and supplemented when needed with data provided from the industrial partners and reported in project deliverables or from literature. The final product yield and level of purity have been also calculated for each scenario.
2. Energy balances, calculated directly at the investigated industrial scale in Superpro Designer. The energy balances were used to calculate the energy consumption per unit of microalgal biomass (kWh kg_DW_^–1^) of each process step.
3. Size of the units, a scale–up/down analysis was performed to investigate the effect of microalgae throughput on the economics of the processing scenarios
4. Breakdown of resources demand in utilities, materials, labour and consumables
5. Cost breakdown into total capital investment (CAPEX) and unit operational costs (OPEX) expressed per kg of processed biomass from cultivation.

##

# Biomass composition for optimised biorefinery scenario in selected strains

The biomass composition of all species were derived as following. Total carbohydrate composition, total protein and total lipids were determined in duplicate (i.e. from 2 cultivation runs). Total carbohydrate composition was based on the total sugar composition as determined by high performance anion exchange chromatography (HPAEC)(de Keijzer et al., 2012). For poly/monosaccharides a 80:20% ratio was estimated. Total protein was determined using Kjeldalh, using species specific N-to-protein ratios. The ratio of soluble and insoluble proteins varies between species. For rapid extraction of total lipid/oil from microalgae the method described by (Axelsson and Gentili, 2014) was used. The method described by (Breuer et al., 2013) was used to determine the fatty acid composition of the lipid/oil fraction. The amount of cell debris was calculated to be the ‘remainder’, e.g. unmeasured biomass.

For (N^-^)-biomass of *N. gaditana* total carbohydrates, total protein and FAME content were determined in duplicate. The absolute amount of ash, cell debris and total proteins were assumed to be constant and linear programming was used to derive the total lipid content, while matching the reported protein% and carbohydrate% of the biomass.

Table S 1 – Biomass composition on a dry basis after cultivation in each value chain

|  | *S. obliquus* | *P. tricornutum* | *I. galbana* | *N. gaditana (N^+^)* | *N gaditana (N^-^)* |
| --- | --- | --- | --- | --- | --- |
| Ash | 7.1% | 18.4% | 12.9% | 9.2% | 4.7% |
| Cell debris | 16.7% | 25.1% | 22.1% | 12.4% | 6.3% |
| Lipids | 12.9% | 21.0% | 22.9% | 30.0% | 60.8% |
| Monosaccharides | 3.5% | 1.2% | 1.3% | 1.6% | 1.5% |
| Pigments | 0.34% | 1.4% | 1.0% | 0.2% | 0.1% |
| Polysaccharides | 14.0% | 4.4% | 5.3% | 6.4% | 6.1% |
| Soluble proteins | 18.3% | 23.9% | 28.4% | 24.2% | 12.3% |
| Insoluble proteins | 27.5% | 6.0% | 7.1% | 16.2% | 8.3% |

# Total Capital Investment, CAPEX and OPEX calculations

Table S 2 – Procedure for calculating Total Capital Investment, CAPEX and OPEX of the biorefineries (excluding cultivation)

| TOTAL CAPITAL INVESTMENT | Direct Costs  (DC) | Major equipment purchase costs | PC |
| --- | --- | --- | --- |
|  |  | Installation costs | 50% PC |
|  |  | Piping | 20% PC |
|  |  | Instrumentation and control | 30% PC |
|  |  | Insulation | 1% PC |
|  |  | Electrical | 10% PC |
|  |  | Buildings | 15% PC |
|  |  | Land improvements | 5% PC |
|  |  | Service facilities | 20% PC |
|  | Indirect Costs (IC) | Construction expenses | 10% DC |
|  |  | Engineering and supervision | 20% DC |
|  | Other Costs (OC) | Contractor's fee | 3% (DC+IC) |
|  |  | Contingency (Major equipment) | 5% (DC+IC) |
|  | Direct fixed capital costs (DFC) | | DC+IC+OC |
|  | Working capital | | OPEX first month of operation |
|  | Total capital investment | | DFC + working capital + Start–up and validation |
| CAPEX | Depreciation | | (DC+IC+OC)/15 years |
|  | Interest | | 8% of depreciation |
|  | Property tax | | 1% DFC |
|  | Insurance | | 0.6% DFC |
|  | Purchase tax | | 1.5% DFC |
| OPEX | Electricity (utility) | | Calculated from equipment consumption |
|  | Other utilities (heating, cooling agents) | | Calculated from equipment mass and energy balances |
|  | Raw materials | | Calculated from mass balances |
|  | Labour | | Salaries + Employer’s contribution |
|  | Supervision | | 20% Labour |
|  | Overheads | | 55% Labour |
|  | Laboratory / Quality Control / Quality Assurance | | 10% Labour cost |
|  | Wastewater treatment | | Calculated from mass balances |
|  | Consumables | | Calculated from equipment design |
|  | Maintenance | | 1% DFC |

# Equipment cost

Table S 3 – Costs of main equipment as a function of a characteristic size, updated to 2015

| CO_2_ adsorption | $\boldsymbol{C}=\boldsymbol{150} € \boldsymbol{m}^{-\boldsymbol{3}}$ | Air volume to process |
| --- | --- | --- |
| Microfiltration | $\mathbf{C}=\mathbf{50} € \mathbf{m}^{-\mathbf{2}}$ | Membrane cost |
|  | $\boldsymbol{C}=\boldsymbol{9}.\boldsymbol{11} \boldsymbol{A}^{\boldsymbol{0}.\boldsymbol{6}}$for $A>0.2m^{2}$ | Unit costs – $\boldsymbol{C}\left[ = \right]\boldsymbol{k}€$ Membrane Area - $A\left[ = \right]m^{2}$ |
| Centrifugation | $\boldsymbol{C}=\boldsymbol{46} \boldsymbol{Q}^{\boldsymbol{0}.\boldsymbol{29}}$for $\boldsymbol{Q}<\boldsymbol{6} \boldsymbol{m}^{\boldsymbol{3}}\boldsymbol{h}^{-\boldsymbol{1}}$ $\boldsymbol{C}=\boldsymbol{28} \boldsymbol{Q}^{\boldsymbol{0}.\boldsymbol{58}}$for $\boldsymbol{6}<\boldsymbol{Q}<\boldsymbol{600} \boldsymbol{m}^{\boldsymbol{3}}\boldsymbol{h}^{-\boldsymbol{1}}$ | Unit costs – $\boldsymbol{C}\left[ = \right]\boldsymbol{k}€$Liquid throughput - $Q\left[ = \right]m^{3}h^{-1}$ |
| High pressure homogenizer | $\boldsymbol{C}=\boldsymbol{27} \boldsymbol{Q}^{\boldsymbol{0}.\boldsymbol{45}}$for $\boldsymbol{Q}>\boldsymbol{0}.\boldsymbol{6} \boldsymbol{m}^{\boldsymbol{3}}\boldsymbol{h}^{-\boldsymbol{1}}$ $\boldsymbol{C}=\boldsymbol{22}$for $\boldsymbol{Q}<\boldsymbol{0}.\boldsymbol{6} \boldsymbol{m}^{\boldsymbol{3}}\boldsymbol{h}^{-\boldsymbol{1}}$ | Unit costs – $\boldsymbol{C}\left[ = \right]\boldsymbol{k}€$Liquid throughput - $\boldsymbol{Q}\left[ = \right]\boldsymbol{m}^{\boldsymbol{3}}\boldsymbol{h}^{-\boldsymbol{1}}$ |
| Spray drying | $\boldsymbol{C}=\boldsymbol{59} \boldsymbol{Q}^{\boldsymbol{0}.\boldsymbol{16}}$for $\boldsymbol{10}<\boldsymbol{Q}<\boldsymbol{10000} \boldsymbol{kg} \boldsymbol{h}^{-\boldsymbol{1}}$ | Unit costs – $\boldsymbol{C}\left[ = \right]\boldsymbol{k}€$Drying capacity - $\boldsymbol{Q}\left[ = \right]\boldsymbol{kg} \boldsymbol{h}^{-\boldsymbol{1}}$ |
| Blending tank | $\boldsymbol{C}=\boldsymbol{153} \boldsymbol{V}^{\boldsymbol{0}.\boldsymbol{14}}$ for $\mathbf{0}.\mathbf{2}<\mathbf{V}<\mathbf{100} \mathbf{m}^{\mathbf{3}}$ | Unit costs – $\boldsymbol{C}\left[ = \right]\boldsymbol{k}€$Tank volume - $\boldsymbol{V}\left[ = \right]\boldsymbol{m}^{\boldsymbol{3}}$ |
| UF/DF | $\boldsymbol{C}=\boldsymbol{50} € \boldsymbol{m}^{-\boldsymbol{2}}$ | Membrane costs |
|  | $\boldsymbol{C}=\boldsymbol{11}.\boldsymbol{7} \boldsymbol{A}^{\boldsymbol{0}.\boldsymbol{49}}$for $\boldsymbol{A}>\boldsymbol{5}\boldsymbol{m}^{\boldsymbol{2}}$ | Unit costs – $\boldsymbol{C}\left[ = \right]\boldsymbol{k}€$Membrane Area - $\boldsymbol{A}\left[ = \right]\boldsymbol{m}^{\boldsymbol{2}}$ |
| Liquid-Solid extractor | $\boldsymbol{C}=\boldsymbol{37} \boldsymbol{V}^{\boldsymbol{0}.\boldsymbol{6}}$for $\boldsymbol{0}.\boldsymbol{1}<\boldsymbol{V}<\boldsymbol{10} \boldsymbol{m}^{\boldsymbol{3}}$ | Unit costs – $\boldsymbol{C}\left[ = \right]\boldsymbol{k}€$Mixer volume - $\boldsymbol{V}\left[ = \right]\boldsymbol{m}^{\boldsymbol{3}}$ |


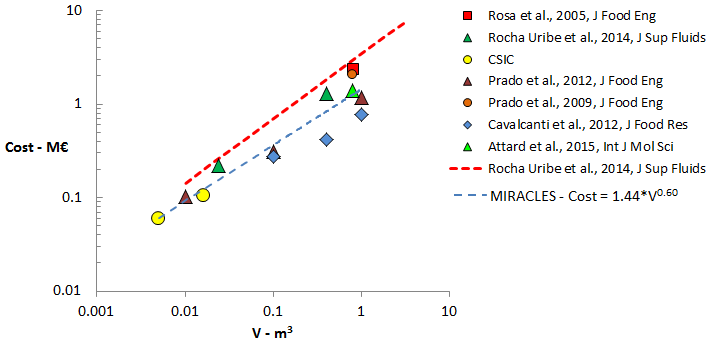


Figure S 11 – Costs of pressurised extraction unit at different industrial scale, updated to 2015

# Price information

Table S 4– Prices for utilities and raw materials

| Item | Price |
| --- | --- |
| Electricity | 0.122 € kWh^‑1^ |
| Heat | 0.0152 € kWh^‑1^ |
| Water | 0.48 € m^‑3^ |
| Water vapor | 0.015 € kg^-1^ |
| Sorbent | 8.00 € kg^-1^ |
| CO_2_ (liquid) | 0.05 € kg^-1^ |
| Ethanol | 0.36 € kg^-1^ |
| Hexane | 0.364 € kg^-1^ |
| Isopropanol | 1.32 € L^‑1^ |
| NaOH | 0.05 € kg^-1^ |
| Enzyme | 2.00 € kg^-1^ |
| HCl | 0.091 € kg^-1^ |

Table S 5 – Foreseeable expected product revenues, as provided by the end-users involved in the MIRACLES project, while taking into account current and expected near-future market sizes (MIRACLES (Multi-product Integrated bioRefinery of Algae: from Carbon dioxide and Light Energy to high-value Specialties), 2017). SP = single product value chain, MP = multiproduct value chain

| Product | Worst case value  **(**€ kg^-1^ product) | Best case value*  **(**€ kg^-1^ product) | Application |
| --- | --- | --- | --- |
| Whole microalgae | 1.50 | 3.00 | Aquaculture feed |
| Broken microalgae | 2.00 | 3.00 | Aquaculture feed |
| Soluble proteins | 4.75 | 8.25 | Soluble proteins with functionality for food/feed |
| Pigments (in oil) | 13.75 | 31.25 | Value based on the composition of the extract, i.e. pigment content |
| Oil | 10.50 | 29.50 | Range from bulk lipids to specialties |
| Polysaccharides | 0.75 | 2.00 | Range from biopolymers to feed/food |
| Peptide rich mix (MP1) | 8.75 | 25.25 | For antimicrobial use in food |
| Peptide rich mix (MP2) | 5.25 | 14.75 | For antimicrobial use in food |
| Peptide rich mix (MP3&4) | 7.75 | 22.25 | For antimicrobial use in food |
| Monosaccharides (with salts) | 0 | 0 | Possible application: fertilizer or biogas |
| Insoluble components (SP-III) | 0 | 3.00 | Best case is based on aquaculture |
| Exhausted biomass (SP-IV) | 0 | 0.50 | Best case is based on aquaculture |
| Exhausted biomass (SP-V) | 0 | 2.00 | Bulk ingredient feed and for material applications |
| Insoluble components (MP1) | 0 | 0.70 | Bulk ingredient feed and for material applications |
| Insoluble components (MP2) | 0 | 0.50 | Bulk ingredient feed and for material applications |
| Insoluble components (MP3) | 0 | 2.00 | Bulk ingredient feed and for material applications |
| Insoluble components (MP4) | 0 | 1.25 | Bulk ingredient feed and for material applications |

** To further refine the near-future market ranges data is required on the price drop for niche products when production further increases. The drop is related to the elasticity of the demand, which is currently still unknown for these products.*

# References

Axelsson, M., and Gentili, F. (2014). A Single-Step Method for Rapid Extraction of Total Lipids from Green Microalgae. *PLOS ONE* 9(2)**,** e89643. doi: 10.1371/journal.pone.0089643.

Baerdemaeker, T.D., Lemmens, B., Dotremont, C., Fret, J., Roef, L., Goiris, K., et al. (2013). Benchmark study on algae harvesting with backwashable submerged flat panel membranes. *Bioresource Technology* 129(0)**,** 582-591. doi: <http://dx.doi.org/10.1016/j.biortech.2012.10.153>.

Bastiaens, L., van Roy, S., Vloemans, S., Vandenbosch, B., Vanhoof, F., Sterckx, h., et al. (Year). "Membrane technology for integrated algae pre-harvesting and medium recirculation", in: *AlgaeBiorefineries for Europe: towards a sustainable economy*).

Bilad, M.R., Vandamme, D., Foubert, I., Muylaert, K., and Vankelecom, I.F.J. (2012). Harvesting microalgal biomass using submerged microfiltration membranes. *Bioresource Technology* 111**,** 343-352. doi: <http://dx.doi.org/10.1016/j.biortech.2012.02.009>.

Breuer, G., Evers, W.A.C., de Vree, J.H., Kleinegris, D.M.M., Martens, D.E., Wijffels, R.H., et al. (2013). Analysis of Fatty Acid Content and Composition in Microalgae. *JoVE* (80)**,** e50628. doi: doi:10.3791/50628.

Brilman, W., Garcia Alba, L., and Veneman, R. (2013). Capturing atmospheric CO2 using supported amine sorbents for microalgae cultivation. *Biomass and Bioenergy* 53**,** 39-47. doi: <https://doi.org/10.1016/j.biombioe.2013.02.042>.

Cavalcanti, R.N., Albuquerque, C.L.C., and Meireles, M.A.A. (2016). Supercritical CO2 extraction of cupuassu butter from defatted seed residue: Experimental data, mathematical modeling and cost of manufacturing. *Food and Bioproducts Processing* 97**,** 48-62. doi: <http://dx.doi.org/10.1016/j.fbp.2015.10.004>.

Coons, J.E., Kalb, D.M., Dale, T., and Marrone, B.L. (2014). Getting to low-cost algal biofuels: A monograph on conventional and cutting-edge harvesting and extraction technologies. *Algal Research* 6, Part B**,** 250-270. doi: <http://dx.doi.org/10.1016/j.algal.2014.08.005>.

Danquah, M.K., Ang, L., Uduman, N., Moheimani, N., and Forde, G.M. (2009). Dewatering of microalgal culture for biodiesel production: exploring polymer flocculation and tangential flow filtration. *Journal of Chemical Technology & Biotechnology* 84(7)**,** 1078-1083. doi: 10.1002/jctb.2137.

de Keijzer, J., van den Broek, L.A.M., Ketelaar, T., and van Lammeren, A.A.M. (2012). Histological Examination of Horse Chestnut Infection by Pseudomonas syringae pv. aesculi and Non-Destructive Heat Treatment to Stop Disease Progression. *PLOS ONE* 7(7)**,** e39604. doi: 10.1371/journal.pone.0039604.

del Pilar Sánchez-Camargo, A., Pleite, N., Herrero, M., Cifuentes, A., Ibáñez, E., and Gilbert-López, B. (2017). New approaches for the selective extraction of bioactive compounds employing bio-based solvents and pressurized green processes. *The Journal of Supercritical Fluids* 128**,** 112-120. doi: <https://doi.org/10.1016/j.supflu.2017.05.016>.

Gerardo, M.L., Oatley-Radcliffe, D.L., and Lovitt, R.W. (2014a). Integration of membrane technology in microalgae biorefineries. *Journal of Membrane Science* 464(0)**,** 86-99. doi: <http://dx.doi.org/10.1016/j.memsci.2014.04.010>.

Gerardo, M.L., Oatley-Radcliffe, D.L., and Lovitt, R.W. (2014b). Minimizing the Energy Requirement of Dewatering Scenedesmus sp. by Microfiltration: Performance, Costs, and Feasibility. *Environmental Science & Technology* 48(1)**,** 845-853. doi: 10.1021/es4051567.

Gerardo, M.L., Van Den Hende, S., Vervaeren, H., Coward, T., and Skill, S.C. (2015). Harvesting of microalgae within a biorefinery approach: A review of the developments and case studies from pilot-plants. *Algal Research* 11**,** 248-262. doi: <http://dx.doi.org/10.1016/j.algal.2015.06.019>.

Gilbert-López, B., Barranco, A., Herrero, M., Cifuentes, A., and Ibáñez, E. (2017a). Development of new green processes for the recovery of bioactives from Phaeodactylum tricornutum. *Food Research International* 99(3)**,** 1056-1065. doi: <http://dx.doi.org/10.1016/j.foodres.2016.04.022>.

Gilbert-Lopez, B., Mendiola, J.A., Fontecha, J., van den Broek, L.A.M., Sijtsma, L., Cifuentes, A., et al. (2015). Downstream processing of Isochrysis galbana: a step towards microalgal biorefinery. *Green Chemistry* 17(9)**,** 4599-4609. doi: 10.1039/c5gc01256b.

Gilbert-López, B., Mendiola, J.A., van den Broek, L.A.M., Houweling-Tan, B., Sijtsma, L., Cifuentes, A., et al. (2017b). Green compressed fluid technologies for downstream processing of Scenedesmus obliquus in a biorefinery approach. *Algal Research* 24**,** 111-121. doi: <https://doi.org/10.1016/j.algal.2017.03.011>.

Günerken, E., D'Hondt, E., Eppink, M.H.M., Garcia-Gonzalez, L., Elst, K., and Wijffels, R.H. (2015). Cell disruption for microalgae biorefineries. *Biotechnology Advances* 33(2)**,** 243-260. doi: <http://dx.doi.org/10.1016/j.biotechadv.2015.01.008>.

Halim, R., Danquah, M.K., and Webley, P.A. (2012). Extraction of oil from microalgae for biodiesel production: A review. *Biotechnology Advances* 30(3)**,** 709-732. doi: <http://dx.doi.org/10.1016/j.biotechadv.2012.01.001>.

Halim, R., Rupasinghe, T.W.T., Tull, D.L., and Webley, P.A. (2014). Modelling the kinetics of lipid extraction from wet microalgal concentrate: A novel perspective on a classical process. *Chemical Engineering Journal* 242**,** 234-253. doi: <http://dx.doi.org/10.1016/j.cej.2013.12.070>.

Ibáñez, E., Gilbert-López, B., Mendiola, J.A., Cifuentes, A., and Herrero, M. (2017). *Integrated green extraction processes for downstream processing of microalgae wet biomass (GREENµWETBIO)*. Spain patent application.

Marcati, A., Ursu, A.V., Laroche, C., Soanen, N., Marchal, L., Jubeau, S., et al. (2014). Extraction and fractionation of polysaccharides and B-phycoerythrin from the microalga Porphyridium cruentum by membrane technology. *Algal Research* 5**,** 258-263. doi: <http://dx.doi.org/10.1016/j.algal.2014.03.006>.

MIRACLES (Multi-product Integrated bioRefinery of Algae: from Carbon dioxide and Light Energy to high-value Specialties) (2017). "Project Final Report", (ed.) <http://miraclesproject.eu/publications.php>.).

Mujumdar, A.S. (2014). *Handbook of Industrial Drying.* CRC Press, Taylor and Francis Group.

Prado, J.M., Assis, A.R., MarÓStica-JÚNior, M.R., and Meireles, M.A.A. (2010). MANUFACTURING COST OF SUPERCRITICAL-EXTRACTED OILS AND CAROTENOIDS FROM AMAZONIAN PLANTS. *Journal of Food Process Engineering* 33(2)**,** 348-369. doi: 10.1111/j.1745-4530.2008.00279.x.

Prado, J.M., Dalmolin, I., Carareto, N.D.D., Basso, R.C., Meirelles, A.J.A., Vladimir Oliveira, J., et al. (2012). Supercritical fluid extraction of grape seed: Process scale-up, extract chemical composition and economic evaluation. *Journal of Food Engineering* 109(2)**,** 249-257. doi: <http://dx.doi.org/10.1016/j.jfoodeng.2011.10.007>.

Rocha-Uribe, J.A., Novelo-Pérez, J.I., and Araceli Ruiz-Mercado, C. (2014). Cost estimation for CO2 supercritical extraction systems and manufacturing cost for habanero chili. *The Journal of Supercritical Fluids* 93**,** 38-41. doi: <http://dx.doi.org/10.1016/j.supflu.2014.03.014>.

Rosa, P.T.V., and Meireles, M.A.A. (2005). Rapid estimation of the manufacturing cost of extracts obtained by supercritical fluid extraction. *Journal of Food Engineering* 67(1–2)**,** 235-240. doi: <http://dx.doi.org/10.1016/j.jfoodeng.2004.05.064>.

Rosa, P.T.V., and Meireles, M.A.A. (2009). "COST OF MANUFACTURING OF SUPERCRITICAL FLUID EXTRACTS FROM CONDIMENTARY PLANTS," in *Extracting Bioactive Compounds for Food Products - Theory and Applications*. (Boca Raton, London, New York: CRC Press), 388-401.

Rossignol, N., Vandanjon, L., Jaouen, P., and Quéméneur, F. (1999). Membrane technology for the continuous separation microalgae/culture medium: compared performances of cross-flow microfiltration and ultrafiltration. *Aquacultural Engineering* 20(3)**,** 191-208. doi: <http://dx.doi.org/10.1016/S0144-8609(99)00018-7>.

Safi, C., Liu, D., Yap, B.J., Martin, G.O., Vaca-Garcia, C., and Pontalier, P.-Y. (2014). A two-stage ultrafiltration process for separating multiple components of Tetraselmis suecica after cell disruption. *Journal of Applied Phycology* 26(6)**,** 2379-2387. doi: 10.1007/s10811-014-0271-0.

Safi, C., Olivieri, G., Campos, R.P., Engelen-Smit, N., Mulder, W.J., van den Broek, L.A.M., et al. (2017). Biorefinery of microalgal soluble proteins by sequential processing and membrane filtration. *Bioresource Technology* 225**,** 151-158. doi: <http://dx.doi.org/10.1016/j.biortech.2016.11.068>.

Show, K.-Y., Lee, D.-J., and Chang, J.-S. (2013). Algal biomass dehydration. *Bioresource Technology* 135(5)**,** 720-729.

Wileman, A., Ozkan, A., and Berberoglu, H. (2012). Rheological properties of algae slurries for minimizing harvesting energy requirements in biofuel production. *Bioresource technology* 104(0)**,** 432-439.

Yu, Q. (2018). *Direct capture of CO2 from ambient air using solid sorbents.* PhD, University of Twente.

Yu, Q., and Brilman, D.W.F. (2017). Design Strategy for CO2 Adsorption from Ambient Air Using a Supported Amine Based Sorbent in a Fixed Bed Reactor. *Energy Procedia* 114**,** 6102-6114. doi: <https://doi.org/10.1016/j.egypro.2017.03.1747>.
